# Uninterruptible Power Supply Improves Precision of Telomere Length Measurement via qPCR

**DOI:** 10.1093/geroni/igaa057.806

**Published:** 2020-12-16

**Authors:** Waylon Hastings, Idan Shalev

**Affiliations:** Pennsylvania State University, University Park, Pennsylvania, United States

## Abstract

Epidemiological literature has produced robust associations between telomere length (TL) and health, wherein individuals with shorter TL are at increased risk for chronic disease and death. Even so, technical challenges associated with TL measurement have led some to question their utility as a biomarker of aging. Several pre-analytical factors influence TL assessment via qPCR including tissue source, sample storage, and DNA extraction method. Additional work has investigated how conditions within the PCR run (e.g. mastermix reagents) influences precision and reproducibility of TL measurements. However, the impact of power supply remains unclear. Momentary fluctuations in power supply can affect the functioning of high-performance electronics, including real-time thermocylers. These fluctuations can be mitigated by using an uninterruptible power source (UPS), an electronic apparatus capable of supplying constant, uninterrupted voltage to high-performance electronics. The current study investigated how using a UPS influenced TL assessment via qPCR. Standard deviation and coefficient of variation across replicates were compared for samples assessed with or without the use of a UPS. Samples run with a UPS had significantly lower standard deviation (p<0.001) and coefficient of variation (p=0.002) than those run without a UPS. Notably, neither the efficiency of exponential amplification (p=0.674) nor the standard curve R2 (p=0.638) varied as a function of UPS status. Thus, UPS status decreases variability within sample replicates while maintaining the overall quality of the qPCR assay. *The work presented was supported by the Telomere Research Network, an NIH-sponsored working-group recently established to coordinate best practices for measuring telomere length in population-based research.

